# Society Meetings

**Published:** 1908-09

**Authors:** 


### SOCIETY MEETINGS.

THE International Congress on Tuberculosis will be held at Washington, beginning September 21, 1908, and continuing until October 12, covering a period of three weeks. The following is a list of special lectures to be delivered and papers to be read in connection with the Congress.

### LIST OF SPECIAL LECTURES.

In connection with the congress a series of special lectures will be delivered in Washington and elsewhere by eminent foreigners. The names of the speakers, and the cities in which they will lecture follow:

Bernard Bang, of Copenhagen, Washington, October 3. Subject: "Studies in Tuberculosis in Domestic Animals and what we may Learn regarding Human Tuberculosis."

A. Calmette, of Lille, France; Philadelphia, September 26. Subject: "Les nouveaux proceeds de diagnostic precoce de la Tuberculosis."

Emil Coni, of Buenos Ayres; Washington, October 2. Subject: "La Lucha contra Tuberculosis en la Republica Argentina."

Arthur Newsholme, of Brighton; Washington, September 29. Subject: "The causes which have lead to the past decline in the death-rate from Tuberculosis and the light thrown by this history on Preventive Action for the Future."

Gotthold Pannwitz, of Berlin; Philadelphia, September 24. Subject: "Social Life and Tuberculosis."

R. W. Philip, Edinburg; Boston, October 6. Subject: "The Antituberculosis Program—Coördination of Preventive Measures."

C. H. Spronck, of Utrecht; Boston, October 7.

Andres Martinez Vargas, of Barcelona; New York, October 9. Subject: "Tuberculosis of the Heart, Blood and Lymph Vessels."

Theodore Williams, of London; Philadelphia, September 25. Subject: "The Evolution of the Treatment of Pulmonary Tuberculosis."

Dr. Maurice Letulle and M. Augustin Rey. (joint lecture); Washington, September 30. Subject: "La Lutte Contra la tuberculose dans les grandes villes par l'Habitation; methodes scientifiques modernes pour sa construction."

Dr. L. Landouzy, of Paris; Baltimore, October 5.

Dr. A. A. Wladimiroff, of St. Petersburg; Washington, September 28. Subject: "Biology of the Bacillus."

Prof. N. Ph. Tendeloo, of Leiden. Subject: "Collateral Tuberculosis Inflammation."

**Titles of Papers for Section I.**

Dr. William H. Welch, president. (Includes titles received up to August 13.)

Milton J. Rosenau, Washington, D. C. "The viability of the tubercle bacillus."

Victor C. Vaughan, Ann Arbor, Mich. "A study of the proteids of the tubercle bacillus."

John Weinzerl, Seattle, Wash. "The action of diffuse light upon bacillus tuberculosis."

Dwight M. Lewis, New Haven, Conn. "The morphology of the tubercle bacillus."

S. Arloing and Paul Courmont, Lyons, France. "Nouvelles Cultures Homogenes des bacilles de la Tuberculose."

J. N. Davalos and J. Cartaya, Havana, Cuba. "Comparative study of the tubercle bacillus of human and of bovine origin."

A. Rodet, Montpelier, France. "La Virulence du bacille dans ses rapports avec l'evolution clinique de la tuberculose pulmonaire."

A Parker Hitchens, Glen Olden, Pa. "A chamber in which dried tubercle bacilli may be handled without danger."

N. Ph. Tendeloo, Leiden, Holland. "Channels of infection."

Julius Bartel, Vienna, Austria. "Uber Eintrittspforten der Tuberkulose."

G. Kuss, Angicourt, France. "Sources et voies d'infection de la contagion tuberculeuse."

S. Bernheim, Paris. "Les portes d'entree de la tuberculose."

S. Bernheim, Paris. "Rapports de l'air avec la contagion tuberculeuse. Sterilisation de l'air."

Alfred F. Hess, New York. "A study of the tuberculous contamination of New York City milk."

Jules Courmont and A. Lesieur, Lyons, France. "Inoculation transcutanee de la tuberculose."

Julius Bartel, Vienna. "Immunisirungsversuche gegen Tuberkulose."

Jules Courmont and A. Lesieur, Lyons. "Contribution a l'immunite dans la tuberculose."

A. B. Marfan, Paris. "Immunite de l'homme pour la tuberculose."

Y. Ishigami, Osaka, Japan. "Tuberculo-toxoidin and immunisation serum."

Eugene L. Opie, New York. "The part of enzymes in tuberculous lesions."

Aldred S. Warthin, Ann Arbor, Mich. "The frequency of healed tuberculosis of the mesenteric glands, with particular reference to the relationship between hyaline deposits in these glands and the healing of tuberculous lesions."

S. Arloing, Lyons, France. "De l'infection tuberculeuse d'apres le criterium anatomo-pathologique."

John McCrae, Montreal, Canada. "Analysis of 1,000 consecutive autopsies in Montreal with reference to the incidence of tuberculosis in the different organs."

A. R. Landry, Montreal, Canada. "Incidence of chronic pleurisy in 1,400 autopsies in Montreal, and its relationship to tuberculosis."

Leon Barnard, Paris. "Etude anatomique et pathologique des lesions non-folliculaires de la tuberculose."

R. Tripier, Lyons. "De la pneumonie dans le processus de la tuberculose pulmonaire."

J. Paviot, Lyons. "Processus anatomique de l'hemorrhagie dans la tuberculose au debut."

Joseph Walsh and C. M. Montgomery, Philadelphia. "The kidneys in tuberculosis of the lungs."

D. J. McCarthy, Philadelphia. "Tuberculosis of the spinal meninges, with a consideration of the mode of infection of these structures."

J. T. Ullon, Philadelphia. "The liver in tuberculosis."

Walter Altschul, Prague, Austria. "Zur patholigie der Peritoneal-tuberkulose."

Charles Esmonet, Puy de Dom. "De la tuberculose experimentale du testicule."

O. Amrein, Arosa, Switzerland. "Periostitis et adipositis tuberculosa toxica multiplex."

Paul Courmont, Lyons. "Proprietes humorales des exsudants tuberculeux, valeur, pronostique et therapeutique."

Camillo Calleja, Valladolid, Spain.

Alfred C. Crofton, Chicago. "An experimental and clinical study of the calcium metabolism in tuberculosis."

---

The American Association of Obstetricians and Gynecologists will hold its twenty-first annual meeting at the Hotel Belvedere, Chase and Charles Streets, Baltimore, Md., Tuesday, Wednesday and Thursday, September 22, 23 and 24, 1908, under the presidency of Dr. E. Gustav Zinke, of Cincinnati. Drs. W. A. B. Sellman (chairman), Joseph H. Branham, and William S. Smith, of Baltimore, constitute the committee of arrangements. Dr. Sellman, whose address is No. 5 East Biddle street, should be communicated with regarding the reservation of rooms at the Belvedere, as well as covers at the banquet.

---

The Mississippi Valley Medical Association will hold its (34th) annual meeting at The Seelbach Hotel, Louisville, Tuesday, Wednesday and Thursday, October 13, 14 and 15, 1908, under the presidency of Arthur R. Elliott, of Chicago. An extensive preliminary program has been issued and a large attendance is expected. Henry Enos Tuley, 111 W. Kentucky street, Louisville, is the secretary, who should be addressed concerning the affairs of the meeting.

